# Direct immunoactivation by chemotherapeutic drugs in cancer treatment

**DOI:** 10.1002/adtp.202300209

**Published:** 2023-09-15

**Authors:** Yurui Wang, Yiran Song, Yazhi He, Yang Wang, Jochen Maurer, Fabian Kiessling, Twan Lammers, Feng Wang, Yang Shi

**Affiliations:** 1 Department of Polymer Therapeutics, Institute for Experimental Molecular Imaging, Uniklinik RWTH Aachen and Helmholtz Institute for Biomedical Engineering, Faculty of Medicine, RWTH Aachen University, Aachen 52074, Germany; 2 Department of Gastroenterology, Shanghai 10th People's Hospital, School of Medicine, Tongji University, Shanghai 200040, PR China; 3 Department of Gynecology and Obstetrics, Uniklinik RWTH Aachen, Aachen 52074, Germany; 4 Institute for Experimental Molecular Imaging, Uniklinik RWTH Aachen and Helmholtz Institute for Biomedical Engineering, Faculty of Medicine, RWTH Aachen University, Aachen 52074, Germany; 5 Department of Gastroenterology, Huadong Hospital, Shanghai Medical College, Fudan University, Shanghai 200040, PR China

**Keywords:** chemotherapeutic drugs, immunoactivation, immune cells, cancer, immunotherapy

## Abstract

The immune system plays a crucial role in recognizing and eliminating pathogenic substances and malignant cells in the body. For cancer treatment, immunotherapy is becoming the standard treatment for many types of cancer and is often combined with chemotherapy. Although chemotherapeutic agents are often reported to have adverse effects, including immunosuppression, they can also play a positive role in immunotherapy by directly stimulating the immune system. This has been demonstrated in preclinical and clinical studies in the past decades. Chemotherapeutics can activate immune cells through different immune receptors and signaling pathways depending on their chemical structure and formulation. In this review, we summarize and discuss the direct immunoactivation effects of chemotherapeutics and possible mechanisms behind these effects. Finally, we prospect chemo-immunotherapeutic combinations for the more effective and safer treatment of cancer.

## 1 Introduction

Conventional anticancer chemotherapy remains the main treatment modality for many types of cancer. Generally, chemotherapeutic agents exert their cytotoxic effects on cancer cells by interrupting metabolic processes, inhibiting DNA replication and interfering with protein biosynthesis or generating chemical species that damage cancer cells.^[1,2]^ For example, cyclophosphamide inhibits DNA synthesis due to its metabolite phosphoramide mustard that induces DNA crosslinking, thus leading to cell death.^[3,4]^ However, the potential contribution of chemotherapeutic agents in activating the immune system is often neglected, ^[5,6]^ and their supportive role in immunotherapy has thus far hardly been exploited. Notably, both in human and animal models, chemical antineoplastic drugs often induce immunosuppression, particularly at high concentration.^[7]^ For example, temozolomide can cause lymphopenia^[8,9]^, methotrexate and cyclophosphamide are used as immunosuppressive agents in the treatment of autoimmune diseases.^[10–12]^

However, an increasing number of preclinical and clinical studies have also shown that chemotherapeutics can provoke cancer immunity.^[13]^ Chemotherapeutics can stimulate the immune system via two main mechanisms. Firstly, certain chemotherapeutic agents directly trigger cancer cells to undergo immunogenic cell death (ICD) which can initiate anti-cancer immune reactions within the tumor microenvironment (TME).^[14]^ During ICD, apoptotic cancer cells express danger-associated molecular patterns (DAMPs) and release cancer antigens which together induce adaptive immunity.^[15,16]^ Moreover, chemotherapeutic agents can also directly activate the immune system, for example, by promoting macrophage polarization, dendritic cell (DC) maturation and T cell response against cancer.^[17–20]^ When M2-like macrophages are polarized into M1-like macrophages by chemotherapeutic drugs, they become pro-inflammatory and produce pro-inflammatory cytokines such as tumor necrosis factor-alpha (TNF-α), interleukin-1β (IL-1β), and interleukin 6 (IL-6).^[21,22]^ Chemotherapeutic drugs can also directly induce DC maturation by promoting the expression of histocompatibility complex (MHC) class molecules, and/or of co-stimulatory molecules such as CD80 and CD86 on the surface of DCs when used at low doses. The maturation of DCs is necessary for presenting antigens and priming T lymphocytes.^[23,24]^ While there is growing evidence supporting the ability of chemotherapeutics to activate the immune system, different chemotherapeutics may induce the same type of immunoactivation via different mechanisms. Moreover, chemotherapeutics can directly contribute to cancer immunotherapy by depleting immunosuppressive cells, including CD4^+^ CD25^+^ regulatory T cells (Tregs) and myeloid-derived suppressor cells (MDSCs).^[25]^ Tregs are known to suppress the immune system and prevent it from attacking cancer cells, while MDSCs also have immunosuppressive functions and inhibit the activation of T cells.^[26,27]^ Studies have reported that certain chemotherapeutic agents, such as gemcitabine and cyclophosphamide, can induce apoptosis and dysfunction of Tregs and MDSCs, thereby enhancing antitumor immune response.^[27–30]^ For example, low-dose gemcitabine has been shown to inhibit tumor growth by depleting master regulator (MR) genes expressing in tumor-infiltrating regulatory T cells (TI-Tregs) *in vivo*.^[31]^

**Figure 1:**
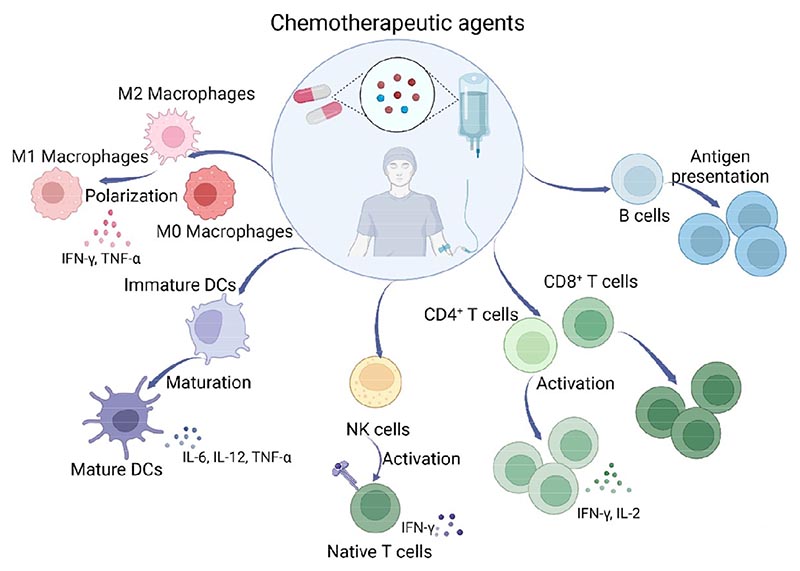
Schematic illustration of direct immunoactivation effects of chemotherapeutic drugs on various immune cells. Chemotherapeutic drugs can directly activate M0 or re-polarize M2-like macrophages to an M1-like phenotype and promote DC maturation. NK cells can be stimulated by chemotherapeutic drugs to be cytotoxic. Chemotherapy has been shown to activate both CD4^+^ and CD8^+^ T cells. Finally, B cells can be stimulated by chemotherapeutics to express activation markers and exhibit enhanced antigen presentation function.

In this review, we provide an overview of the immunoactivation effects of chemotherapeutic drugs (Figure 1). We identify prerequisites for achieving optimal combinations of chemo- and immunotherapy, and discuss the molecular and cellular mechanisms through which conventional chemotherapeutics agents directly activate immune cells such as macrophages, DCs, NK cells, T cells and B cells. This information may guide clinicians and researchers to develop effective immunotherapeutic strategies that harness the synergistic effects of chemotherapy and immunotherapy.

## 2 Direct activation of innate immune system by chemotherapeutic agents

The innate immune system is the first line of defense against invading pathogens and is an essential part of the body's immune response.^[32]^ The innate immune system is composed of physical, chemical and cellular barriers.^[33]^ Skin and mucous membranes are part of the innate immune system which act as a physical barrier to prevent pathogens from entering the body.^[34,35]^ Chemical barriers include enzymes and proteins, such as lysozyme and complement, which can break down and destroy foreign invaders. Cellular barriers include various types of immune cells, such as macrophages, neutrophils, and NK cells, which can recognize and eliminate pathogens through phagocytosis, releasing of toxic substances, or inducing apoptosis.^[36,37]^ Unlike the adaptive immune system, which relies on the recognition of specific antigens, the innate immune system can recognize a wide range of pathogens with pattern recognition receptors (PRRs) that recognize conserved structures of microbial components, called pathogen-associated molecular patterns (PAMPs). The innate immune system provides rapid and non-specific defense against invading pathogens and plays a critical role in preventing infections and maintaining homeostasis within the body.^[36,38]^ It is also essential in the initiation of anti-cancer immunity.

### 2.1 Chemotherapeutic agents polarizing macrophages

Macrophages play crucial roles in host defense against tumors and stimulate antitumor immune response.^[39]^ Macrophages originate from monocytes and are further differentiated into subtypes, for example, conventionally defined type 1 pro-inflammatory (M1) and type 2 anti-inflammatory (M2) subsets. The M1 subset is classically activated macrophages contributing to anti-infection and antitumor effects, ^[40]^while the M2 subset is alternatively activated and responsible for tissue repairment.^[41–43]^ The presence of immunomodulatory factors such as interferon-γ (IFN-γ), IL-4, IL-10, and transforming growth factor-beta (TGF-β) can significantly impact macrophage function and phenotype.^[44]^ Several studies have investigated that chemotherapeutic regimens were able to subvert the pro-tumorigenic activities of macrophages and increase the production of inflammatory cytokines by switching from an M2-like phenotype to an M1-like phenotype in vivo.^[45,46]^ For example, paclitaxel, the active component of Taxol, is an important antineoplastic drug widely used against a variety of solid tumor types^[47]^ by disrupting cellular microtubule networks.^[48]^ Low-dose paclitaxel treatment induced the skewing of M2-like macrophages into M1-like macrophages, while it did not exert obvious cytotoxicity to macrophages.^[49,50]^ Furthermore, emerging evidence indicates that paclitaxel's most significant immune activity is its capacity to reverse macrophage-mediated immune suppressor activities.^[51]^

Several reports have described that paclitaxel has lipopolysaccharide (LPS)-mimetic capabilities to reprogram M2-polarized macrophages to M1 through the Toll-like receptor-4 (TLR-4) without inducing their apoptosis.^[52–54]^ TLR-4 is expressed on various immune cells as cell-surface receptors to recognize pathogens and mediate inflammatory response.^[49]^ TLR-4 is the most important member of the TLR protein family for LPS recognition and its ligation leads to the activation of the TNF-α signaling pathway as well as the production of various inflammatory cytokines.^[27,55,56]^ Cullis et al. have shown that the antitumor activity of nab-paclitaxel, an albumin-based nanoformulation of paclitaxel, may be TLR-4-dependent because TLR-4 inhibitors abrogated immunostimulatory cytokine expression by macrophages *in vivo* and vitro.^[51]^ Therefore, the polarization of M2-like macrophages to an M1-like phenotype by paclitaxel may occur by the activation of TLR-4 signal pathway. Micropinocytosis of nab-paclitaxel promotes macrophage polarization may account for its elevated drug delivery ability over the conventional solvent-based formulation of paclitaxel.^[57]^ Furthermore, Kashyap et al. reported that microtubule-targeting drug, plinabulin, can directly promote M1-Like macrophage polarization and proliferation of macrophages via the Jun N-terminal kinase pathway both in murine and human models..^[58]^

Ding and colleagues sought to identify the underlying mechanism of Taxol immunoactivation.^[59]^ The myeloid differentiation protein-88 (MyD88) is a crucial signal adaptor protein required for binding the cognate ligand to active TLR-4. MyD88 signaling activates downstream molecules such as mitogen-activated protein kinases (MAPKs) and nuclear factor-κB (NF-κB).^[60,61]^ To determine whether TLR-4 and its downstream molecule MyD88 are required for the immunoactivation effects of paclitaxel and LPS, the authors used primary macrophages from TLR-4-deficient or MyD88-knockout mice. Macrophages from wild-type mice were shown to secrete tumor necrosis factor (TNF) and nitric oxide (NO) after treatment with paclitaxel, whereas macrophages from TLR4-deficient or MyD88-knockout mice produced significantly lower levels of these two soluble factors. Furthermore, paclitaxel did not induce MAPK and NF-κB activation in TLR4-deficient macrophages and delayed NF-κB activation in MyD88 knockout macrophages. These results suggest that paclitaxel and LPS may share TLR4/MyD88-dependent and TLR4-dependent/MyD88-independent pathways in the production of inflammatory mediators.

**Figure 2:**
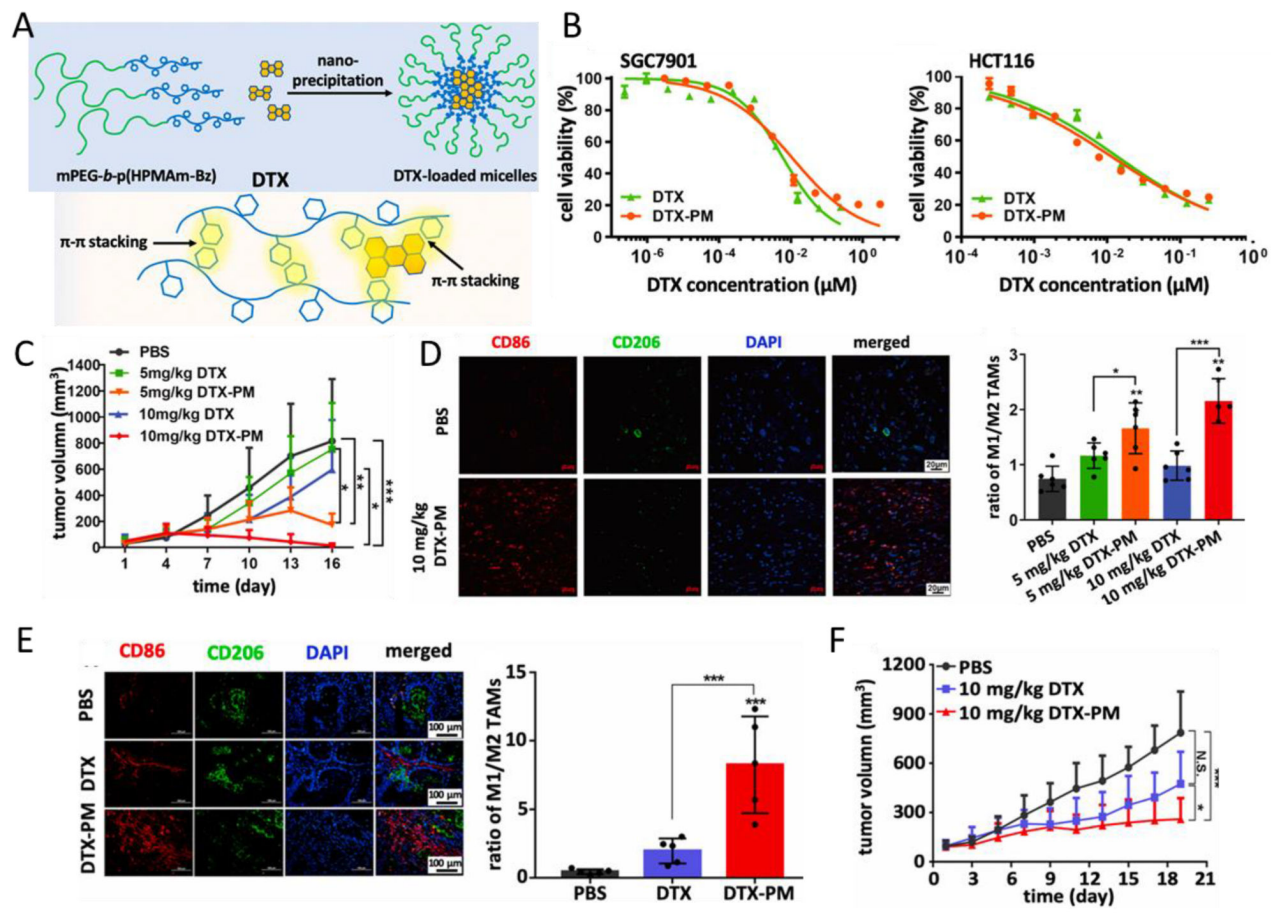
Π electron-stabilized polymeric micelles loaded with DTX (docetaxel) for tumor treatment and macrophage polarization. A) DTX (docetaxel) was incorporated into PM (polymeric micelles) formulation based on mPEG-b-p(HPMAm-Bz) by a nanoprecipitation method. B) The IC50 of both free docetaxel and DTX-PM (polymeric micelles loaded with docetaxel) in two cell lines was determined. C) Tumor growth curves of mice treated with PBS, docetaxel, and polymeric micelles loaded docetaxel at 5 and 10 mg/kg in a cell line-derived subcutaneous xenografts model. D) The polarization of TAM (tumor-associated macrophages) was assessed through immunofluorescence staining and the ratio between M1-like macrophages (CD86 staining; red) and M2-like TAM (CD206 staining; green) was calculated, revealing a significantly higher presence of M1-like TAM following treatment with polymeric micelles loaded docetaxel. E) Tumor growth curves of mice treated with PBS, docetaxel, and polymeric micelles loaded docetaxel at 10 mg/kg in a patient-derived xenograft (PDX) model. F) Immunofluorescence microscopy analysis of PDX tumors demonstrated a significantly increased ratio of M1/ M2-like macrophages following treatment. Reproduced with permission.^[66]^ Copyright 2021, Elsevier.

Recently, nanomedicines have been employed to deliver immunomodulatory chemotherapeutic drugs.^[48,62]^ Nanomedicines can overcome certain physiological barriers and achieve enhanced tumor drug accumulation via the enhanced permeability and retention (EPR) effect, while at the same time attenuating adverse effects of drugs in healthy tissues.^[63–65]^ We have previously demonstrated the ability ofdocetaxel-loaded Π electron-stabilized polymeric micelles to polarize macrophages to an M1-like phenotype. The micelle formulation exhibited high stability and effectively incorporated docetaxel via Π-Π stacking interactions (Figure 2A).^[66]^ In vitro cytotoxicity studies showed that polymeric micelles loaded with docetaxel were equally efficient as free docetaxel in inhibiting gastrointestinal (GI) cancer cell growth in vitro (Figure 2B). Then, multiple mouse models included cell-line derived xenografts (CDX) and patient-derived xenografts (PDX) were established to investigate the effectiveness of polymeric micelles loaded docetaxel in advanced-stage GI cancer. The efficacy of the polymeric micelles loaded with docetaxel was evaluated using a subcutaneous CDX model of SGC7901 cells. Free docetaxel slightly inhibited tumor growth while the polymeric micelles loaded with docetaxel showed significantly higher efficacy at two dose levels (5 and 10 mg/kg). The better efficacy was achieved at a dose of 10 mg/kg, leading to nearly complete tumor regression after repeated injection, whereas free docetaxel at the same dose was less effective (Figure 2C). In addition to its antitumor effect, immunofluorescence staining was performed to identify M1-like and M2-like macrophages. Compared to the non-treated group, the ratio of M1-like to M2-like macrophages was significantly increased in mice treated with free docetaxel and polymeric micelles loaded with docetaxel. Importantly, the micelle formulation of docetaxel showed more effective macrophage polarization than free docetaxel (Figure 2D). Furthermore, in a PDX model, the polymeric micelles loaded with docetaxel showed a significantly increased ratio of M1-/M2-like macrophages (Figure 2E) and better therapeutic efficacy compared to free docetaxel (Figure 2F). This work demonstrated that Π electron-stabilized polymeric micelles improve the therapeutic efficacy and macrophage modulation effect of docetaxel.

Several clinical trials have provided evidence that doxorubicin can provoke cancer immunity by promoting macrophage polarization from an M2-like to M1-like phenotype through TLR-4 pathway. Notably, blocking TLR-4 signaling effectively decreased doxorubicin-induced macrophage polarization.^[67–69]^ Similarly, low-dose cyclophosphamide induced re-polarization of M2-like macrophages to a more M1-like phenotype in vivo, upregulating the production of the pro-inflammatory cytokines IL-6 and IL-12, while down-regulating the anti-inflammatory cytokines IL-10 and TGF-β.^[70]^ In another study, formyl peptide receptor (FPR1/2) modified cisplatin prodrug was reported to active macrophages by delivering cisplatin to FPR1/2-overexpressing immune cells including macrophages and triggered the production of proinflammatory cytokines such as TNF-α and IFN-γ.^[71]^

### 2.2 Direct activation of dendritic cells by chemotherapeutic agents

DCs are antigen-presenting cells (APCs) and crucial for the initiation of antitumor T-cell response. ^[25,26]^ Upon antigen capture, DCs undergo a maturation process in which they upregulate the expression of MHC molecules, costimulatory molecules (CD80/86), cytokines, chemokines, and the chemokine receptor CCR7. ^[27,28]^ Consequently, mature DCs migrate to regional lymphoid tissues via CCR7 and prime naive T cells to differentiate to various effector T cells, including T helper (Th) 1 cells, Th2 cells, Th17 cells, T follicular helper (TFH) cells, and CD8^+^ cytotoxic T-lymphocytes (CTLs) ^[19,27,28]^. Activation of naive DCs is crucial to trigger antigen-specific immune response and elicit anti-cancer effects.

**Figure 3:**
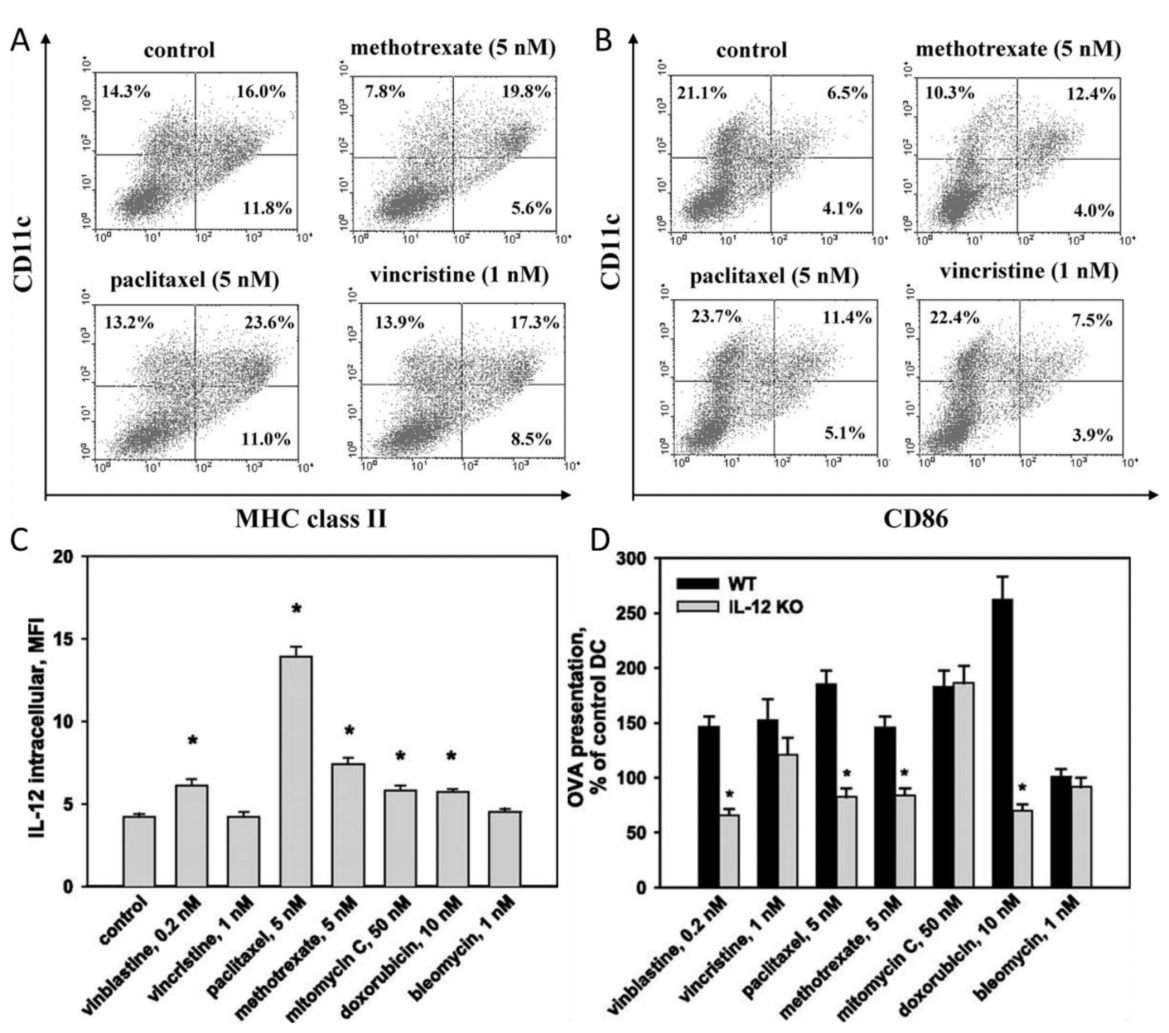
Activation of DCs by anticancer chemotherapeutic agents. The chemotherapeutic agents were added to the cultures at the specified concentrations for 48 hours. A) Flow cytometry plots of CD11c^+^ MHC-II^+^ DCs. B) Percentages of CD11c^+^ CD86^+^ DCs. C) Differentiated DCs were treated with specific concentrations of cytotoxic drugs for 48 hours and then intracellular staining for IL-12 was performed without any additional DC activators. D) The levels of ovalbumin (OVA) presentation by DCs from wild-type and IL-12 knockout mice after pretreatment with chemotherapeutic agents were compared. Reproduced with permission.^[77]^ Copyright 2009, American Association of Immunologists.

Maturation of DCs is a complex process, encompassing multiple functions, including antigen processing and presentation, migration to lymphoid tissues, and T cell priming. These functions bridge innate and adaptive immunity.^[72–74]^ Recently, studies have suggested that several chemotherapeutic agents may directly affect DC maturation.^[75,76]^ Shurin et al. reported that a variety of chemotherapeutic agents at low nontoxic concentrations can upregulate the antigen processing machinery and enhance CD80, CD40 and IL-12p70 expression in murine bone marrow-derived DCs, ^[77]^ these costimulatory molecules are crucial to T cell activation.^[73,78]^ Furthermore, since MHC class complexes are important in T cell priming,^[79,80]^ paclitaxel and methotrexate could increase percentages of CD11c^+^ CD86^+^ and CD11c^+^ MHC-II^+^ DCs for more efficient T cell activation (Figure 3A and B). In addition, it was shown that chemotherapeutics drugs, especially paclitaxel, can increase IL-12 expression by DCs (Figure 3C). All tested chemotherapeutic drugs up-regulated antigen presentation in wild-type DCs, however, the ability of paclitaxel, methotrexate, doxorubicin, and vinblastine to upregulate antigen presentation was significantly abrogated in IL-12 knock-out mice (Figure 3D).

CD40 expression on DCs is critical for the induction of proliferation of allogeneic T lymphocytes and Th1 response.^[81,82]^ Akira et al. revealed that chemotherapeutic agents, for example vinblastine, could increase the expression of CD40, CD80, CD86, and MHC II, and trigger IL-1?, IL-6, and IL-12 production by mouse bone marrow-derived DCs.^[83]^ Notably, local injection of low concentrations of vinblastine could induce phenotypic and functional maturation of DCs by triggering in situ maturation of skin-resident DCs.^[84,85]^ Gruijand colleagues found that short-time exposure to anthraquinone-derivatives such as mitoxantrone and doxorubicin promoted the differentiation of CD34^+^ DCs precursors into mature DCs, which then induced primary allogeneic T cell proliferation.^[86]^

The mechanisms of DC differentiation and maturation by chemotherapeutic drugs remain to be fully elucidated. The most extensively studied family of pattern-recognition receptors (PRRs) on DCs are the TLRs.^[87]^ Similar to macrophage polarization, three major signaling pathways determine the function of TLR activation on DCs: NF-κB, MAPK and interferon regulatory factor (IRF).^[88,89]^ TLRs stimulate different Toll/IL-1R homology (TIR) domain signaling molecules, such as MyD88, interferon-β (TRIF), TRIF-related adaptor molecule (TRAM) and TIRAP (MAL), and most TLR ligation activates TAK1 (TGF-β Activated Kinase 1; MAP3K7) by utilizing the MyD88 adaptor pathway. This activates downstream signals such as NF-κB and MAPK, and then induce pro-inflammatory cytokines secretion (TNF-α, IL-12 and IL-6).^[90–92]^ It has been proposed that low dose paclitaxel can induce DC maturation by interacting with TLR-4.^[93,94]^ Recent studies have shown that activation of TLR-4 on dendritic cells induced release of pro-inflammatory mediators, eventually contributing to antibody response.^[95,96]^ Armstrong and colleagues found that low dose paclitaxel enhanced DC maturation and upregulates the expression of costimulatory molecules including CD80 and CD86 by signaling through TLR-4. Blocking of the TLR-4 signaling pathway inhibited the expression of co-stimulatory molecules by 50%.^[97]^

**Figure 4:**
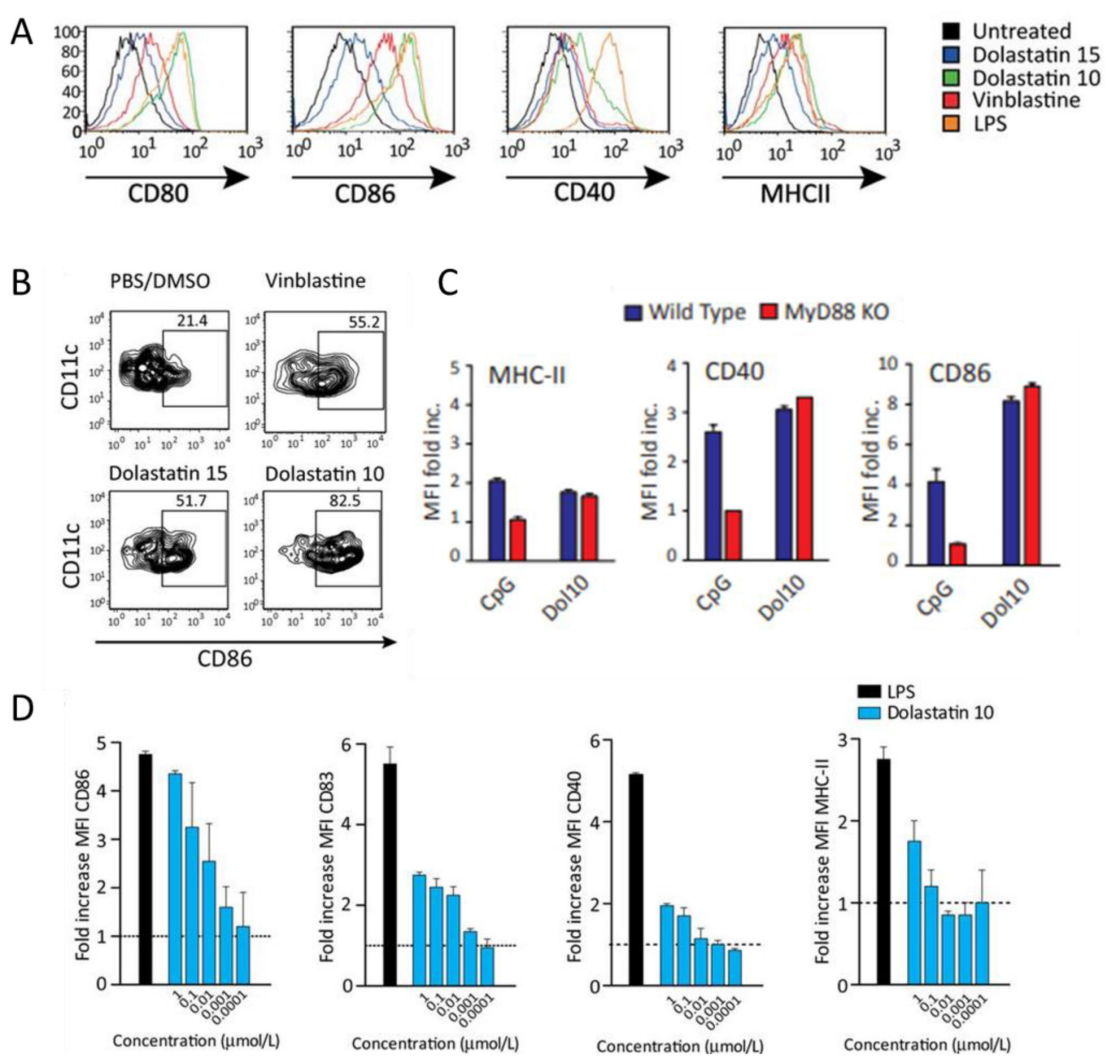
Dolastatins induce phenotypic and functional DC maturation in vitro and in vivo. A) Representative histograms from experiments showing the expression levels of MHC-II and costimulatory molecules CD80, CD86, and CD40 on SP37A3 murine DCs after treatment. B) Dolastatin 10, dolastatin 15, vinblastine or vehicle alone were injected into ears of C57BL/6 mice. After 24 hours, ear skin specimens were collected and cells were then stained with antibodies against CD45, CD11c, MHC-II, and CD86 for flow cytometry analysis. C) Expression of MHC-II, CD40 and CD86 by BM-DCs in wild-type (C57BL/6) and MyD88 knock-out mice was evaluated after treatment with dolastatin 10 or CpG 1668 for 24 hours. D) Treatment of human DCs with dolastatin 10 led to the upregulation of co-stimulatory molecules and enhanced T cell priming. Reproduced with permission.^[101]^ Copyright 2014, American Association for Cancer Research.

In addition to traditional chemotherapeutic drugs, dolastatin, a family of natural microtubule inhibitors isolated from Dolabella Auricularia, has also been shown to induce DC migration and maturation.^[98,99]^ Dolastatin 15 is a seven-subunit depsipeptide and dolastatin 10 is a five-subunit peptide obtained from the same organism.^[100]^ Müller et al. demonstrated that dolastatin upregulates the maturation markers CD80, CD86, CD40 and MHC-II expression by murine DCs (Figure 4A).^[101]^ Consistent with the in vitro results, dolastatin 10 and 15 induced the expression of CD86 and MHC-II on skin-resident langerhans cells (LCs) in C57BL/6 mice (Figure 4B). Interestingly, dolastatin induced similar levels of CD86, CD40 and MHC-II expression by DCs from wild-type and MyD88^-^/^-^ mice while CpG provoked lower expression of these markers by DCs from MyD88^-^/^-^ mice than wild-type DCs. This result suggested that the DC activation effects of dolastatin were MyD88-independent (Figure 4C). Similar responses were observed in human monocyte-derived DCs (Figure 4D). These results suggested that dolastatin-based therapies can activate DCs, thus, contributing to anti-cancer immunity.

### 2.3 NK cell activation by chemotherapy

NK cells are a type of cytotoxic lymphocytes that are part of the innate immune system, displaying cytotoxic functions and producing several cytokines.^[7,102]^ The cytotoxic and immunomodulatory activities of NK cells can be influenced by chemotherapeutic drug administration. In fact, chemotherapy regimens used to treat cancer patients mainly work by inhibiting NK cell-mediated killing of cancer cells and their production of cytokines.^[103,104]^ However, several experiments analyzing the effects of chemotherapeutic drugs on NK cells have shown that changing the type and dose of the drug can have different effects. Recently, a low-dose metronomic cyclophosphamide regimen was reported to stimulate NK-dependent antitumor immunity and promote cytokine production, reducing tumor angiogenesis through Treg depletion.^[25]^ Incubation of human NK cells with paclitaxel resulted in a dose-dependent reduction in cytotoxicity against tumor cell lines, as well as peripheral blood mononuclear cells (PBMCs), MHC-unrestricted T cells and CTLs.^[25]^ There is much evidence about the ability of NK cells to reduce the progression of multiple myeloma. Santoni et al. reported that myeloma cells treated with low doses of doxorubicin, melphalan, and bortezomib up-regulate DNAX accessory molecule-1 (DNAM-1) and NKG2D ligands resulting in enhanced NK cell susceptibility.^[105]^ Additionally, tumors treated with 5-fluorouracil and IFN-α increasingly expressed the MHC-I and presented with higher numbers of infiltrating NK cells. This indicates that NK cells can be activated by 5-fluorouracil and IFN-α treatment.^[104,106]^

## 3 Direct activation of adaptive immune system by chemotherapeutic agents

Adaptive immunity is crucial when innate immunity cannot effectively eliminate infection.^[107]^ The main immune cells of the adaptive immune system are T and B lymphocytes. T cells are activated by T cell receptor interaction with MHC class I/II molecules, co-stimulation signals, and cytokines. This activation process stimulates T cell differentiation, primarily into CD8^+^ cells, Th CD4^+^ cells, and Tregs.^[108,109]^ B cells are stimulated mainly by B cell receptors and CD40, and differentiate into antibody-producing cells, i.e., plasma cells.^[81,110]^

### 3.1 Chemotherapeutic agents activating cytotoxic and helper T cells

High-dose chemotherapeutics can exhibit cytotoxic effects on T lymphocytes, leading to prolonged immunosuppression.^[111,112]^ In contrast, several recent studies demonstrated that chemotherapeutic agents displayed positive effects on T cells and improved T cell-mediated immune response.^[113]^ For example, Tseng et al. found that tumor-bearing mice treated with cisplatin followed by CRT/E7 (calreticulin linked to human papillomavirus type 16 E7 antigen) DNA vaccine exhibited an increased E7-specific CD8^+^ T cell response, leading to effective antitumor response and long-term survival.^[114]^ In another study, low-dose cisplatin and paclitaxel reduced immunosuppression and induced strong tumor-specific CD8^+^ T cell response in both murine and patients.^[115]^ Kim, Daejin and colleagues demonstrated that doxorubicin enhanced proliferation of antigen-specific CD4^+^ and CD8^+^ T cells when co-administered with Ii-PADRE (invariant Pan MHC-II cell surface receptor reactive epitope) DNA vaccination.^[116]^ Moreover, treatment with 5-fluorouracil also increased production of IFN-γ by tumor-specific CD8^+^ T cells within the tumor and promoted T cell-dependent antitumor response.^[117]^ Additional studies demonstrated that docetaxel can elevate IFN-γ production by CD8^+^ T cells and potentiate antigen-specific T-cell response.^[118]^

Naive CD4^+^ T cells can be differentiated into Th cells,^[119]^ including Th1, Th2, Th17 cells based on their cytokine profiles.^[120,121]^ Additionally, Th cells exhibit high plasticity, meaning that cells from one lineage can convert into another lineage under specific conditions. Cyclophosphamide is an alkylating agent widely employed in chemotherapy. Apart from its direct cytotoxic effects on cancer cells, cyclophosphamide exerts direct immunomodulatory effects on T cells.^[122]^ Various studies showed that treatment with cyclophosphamide followed by the adoptive transfer of tumor-reactive CD4^+^ T cells led to the eradication of pleural mesothelioma tumors.^[123]^ These studies have demonstrated that the robust antitumor effects are associated with the development of Th1 antitumor immunity. Cyclophosphamide has been found to induce a shift from a Th2 to a Th1 cytokine profile in lymphoma-bearing mice, promoting the differentiation of CD4^+^ T cells into highly activated effector cells.^[124,125]^ These effector cells have the ability to produce multiple Th1-type cytokines such as IL-2, IFN-γ, and TNF-α simultaneously.

**Figure 5:**
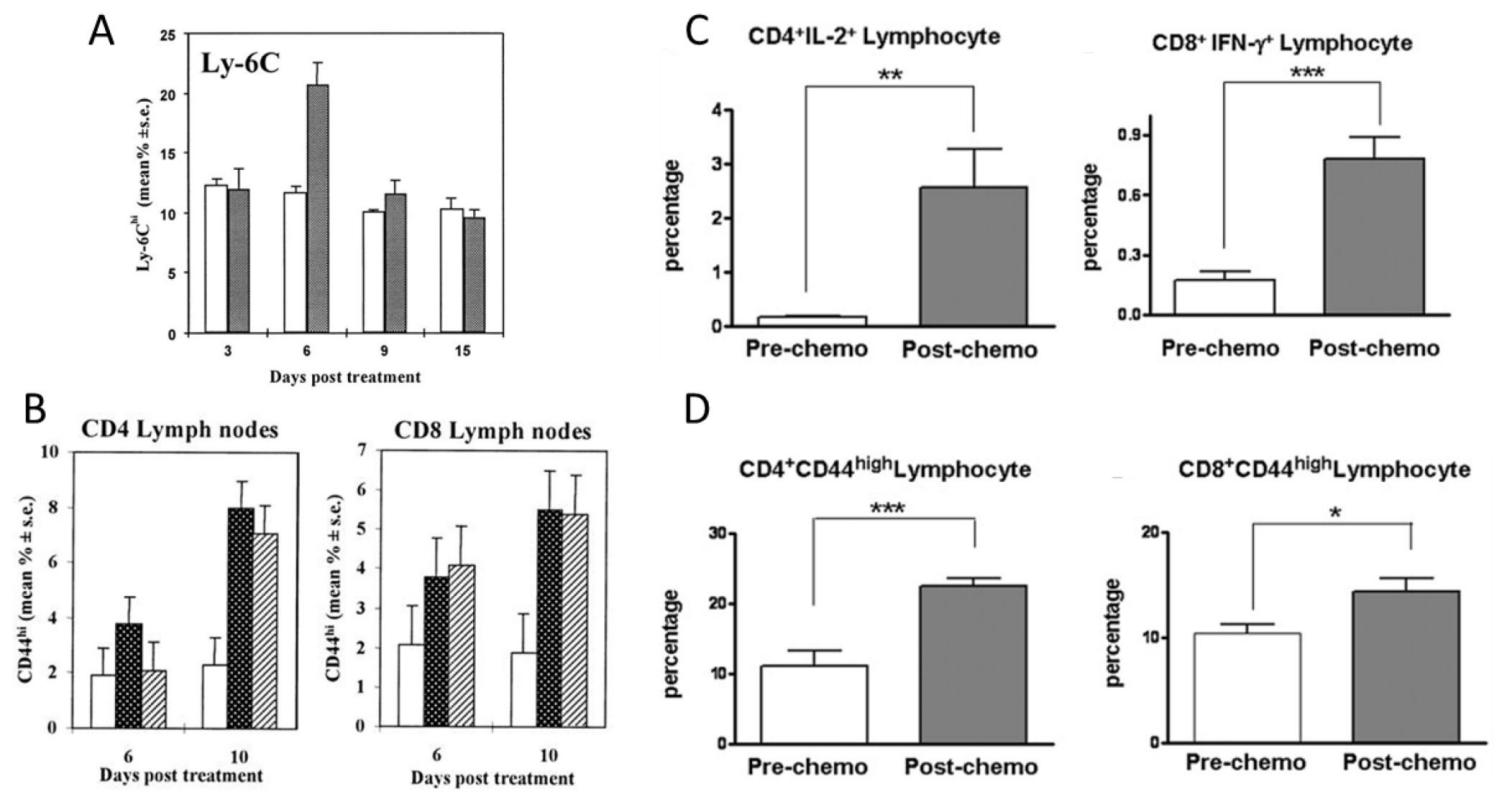
T cell activation effects of cyclophosphamide and paclitaxel. A) Percentages of Ly-6C positive T cells in mice treated with cyclophosphamide (grey bars) and saline (white bars). B) Increase in the percentage of CD44hi T lymphocytes in the CD4^+^ and CD8^+^ T cells from lymph nodes of mice after treated by cyclophosphamide (black bars), poly I:C (dashed bars) and saline (white bars). Reproduced with permission.^[126]^ Copyright 2000, Elsevier. C) Increase of CD4^+^ T cell expressing intracellular IL-2^+^ (left panel) and CD8^+^ T cell expressing intracellular IFN-? (right panel) in NSCLC patients before and 5 days after paclitaxel-based chemotherapy. D) The percentage of CD44^high^ cells on CD4^+^ T cells and CD8^+^ T cells (right panel) from PBMCs and stained for CD44 before and 5 days after chemotherapy. Reproduced with permission. ^[128]^ Copyright 2008, Elsevier.

Proietti and colleagues discovered that low-dose cyclophosphamide increased the number of CD44^high^ T cells in mice.^[126]^ They also found a significant increase Ly-6C positive T cells in the spleen of mice on different days after treatment with a single injection of cyclophosphamide (Figure 5A). The percentages of CD44^high^ T cells in the spleen and lymph nodes of mice treated with cyclophosphamide and poly I:C (a TLR-3 agonist) were found to be higher compared to that of mice injected with saline. In poly I:C injected mice, a similar increase in the percentages of CD44^high^ T cells was observed as compared to that of the cyclophosphamide-treated group (Figure 5B). These results suggest that both the injection of cyclophosphamide and poly I:C led to T cell activation. Moreover, type I IFN in the serum, spleens and lymph nodes was also upregulated by cyclophosphamide treatment in mice.^[126,127]^ These findings indicate that cyclophosphamide can promote the CD44^high^ phenotype in both CD4^+^ and CD8^+^ T cells. Besides, Xiong et al. demonstrated that paclitaxel can augment Th1 cytokine (IFN-γ and IL-2) production in patients with advanced non-small cell lung cancer.^[128]^ Flow cytometry data showed a significantly increased number of CD4^+^ IL-2^+^ T cells and CD8^+^ IFN-γ^+^ T cells among total lymphocytes after paclitaxel treatment (Figure 5C). High expression of CD44 on T cells indicates T cell actitation and the CD44 expression levels were elevated for both CD4^+^ T cells and CD8^+^ T cells in mice treated with paclitaxel (Figure 5D).

**Figure 6:**
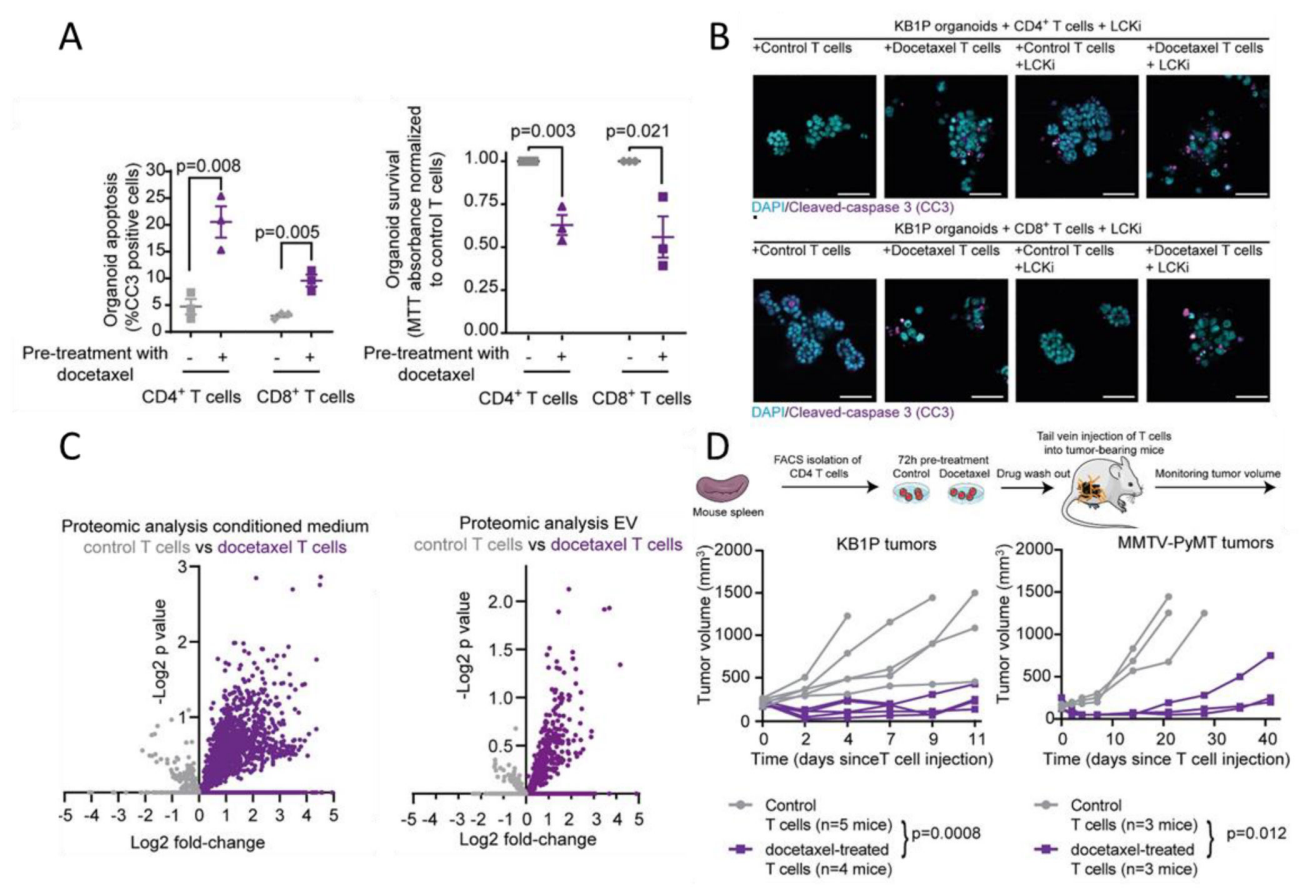
Docetaxel treatment induces T cell cytotoxicity by releasing extracellular vesicles. A) Quantification of cleaved caspase 3 (CC3) and MTT assay assessment of KB1P organoids after co-culturing with either CD4^+^ or CD8^+^ T cells pre-treated with docetaxel or a vehicle. B) Immunofluorescent staining of KB1P and MMTV-PyMT organoids co-cultured with either CD4^+^ or CD8^+^ T cells pre-treated with docetaxel or a vehicle. An LCKi was added to inhibit TCR-mediated killing. C) Volcano plot of proteins in the media (left) containing EVs (right) derived from T cells treated with a vehicle (gray) or docetaxel (purple). D) Growth curves of KB1P tumors (left) or MMTV-PyMT tumors (right) in mice injected with CD4^+^ T cells pre-treated with docetaxel or a vehicle in vitro. Reproduced under terms of the CC-BY license.^[128]^ Copyright 2023, The Authors, published by Wiley.

Recently, Vennin and colleagues reported that chemotherapeutics such as taxanes can trigger tumor cells eradicated by directly increase T cell cytotoxic activity without T cell receptors (TCR) activation both in murine and human models.^[129]^ CD4^+^ and CD8^+^ T cells were isolated from mouse spleens and treated with docetaxel in vitro, subsequently, these treated T cells were co-cultured with organoids derived from tumor suppressor genes Brca1 and Trp53 (KB1P) models. These T cells induced noticeably higher cell death in the organoids compared to untreated T cells (Figure 6A). To investigate whether the T cell killing was mediated by TCR, a lymphocyte kinase inhibitor (LCKi) was used to block TCR-mediated killing. The LCKi didn't hinder docetaxel-induced T cell cytotoxicity in the KB1P organoids (Figure 6B). Interestingly, docetaxel-treated T cells contained a higher protein content compared to non-treated T cells. Notably, these proteins did not belong to known factors driving T cell-mediated killing. A gene ontology analysis revealed high quantities of proteins associated with extracellular vesicles (EVs) derived from docetaxel-treated T cells and the result was confirmed by a proteomics analysis (Figure 6C). Splenic T cells from healthy mice were treated with docetaxel and then intravenously injected into mice with KB1P or MMTV-PyMT (overexpression of the oncogene PyMT) tumors. The mice receiving docetaxel-treated T cells displayed significantly slower tumor growth compared to those receiving non-treated T cells (Figure 6D). These results suggest that docetaxel-treated T cells have a potent anti-cancer effect via a TCR-independent mechanism.

### 3.2 Chemotherapy-induced activation of B cells

**Figure 7:**
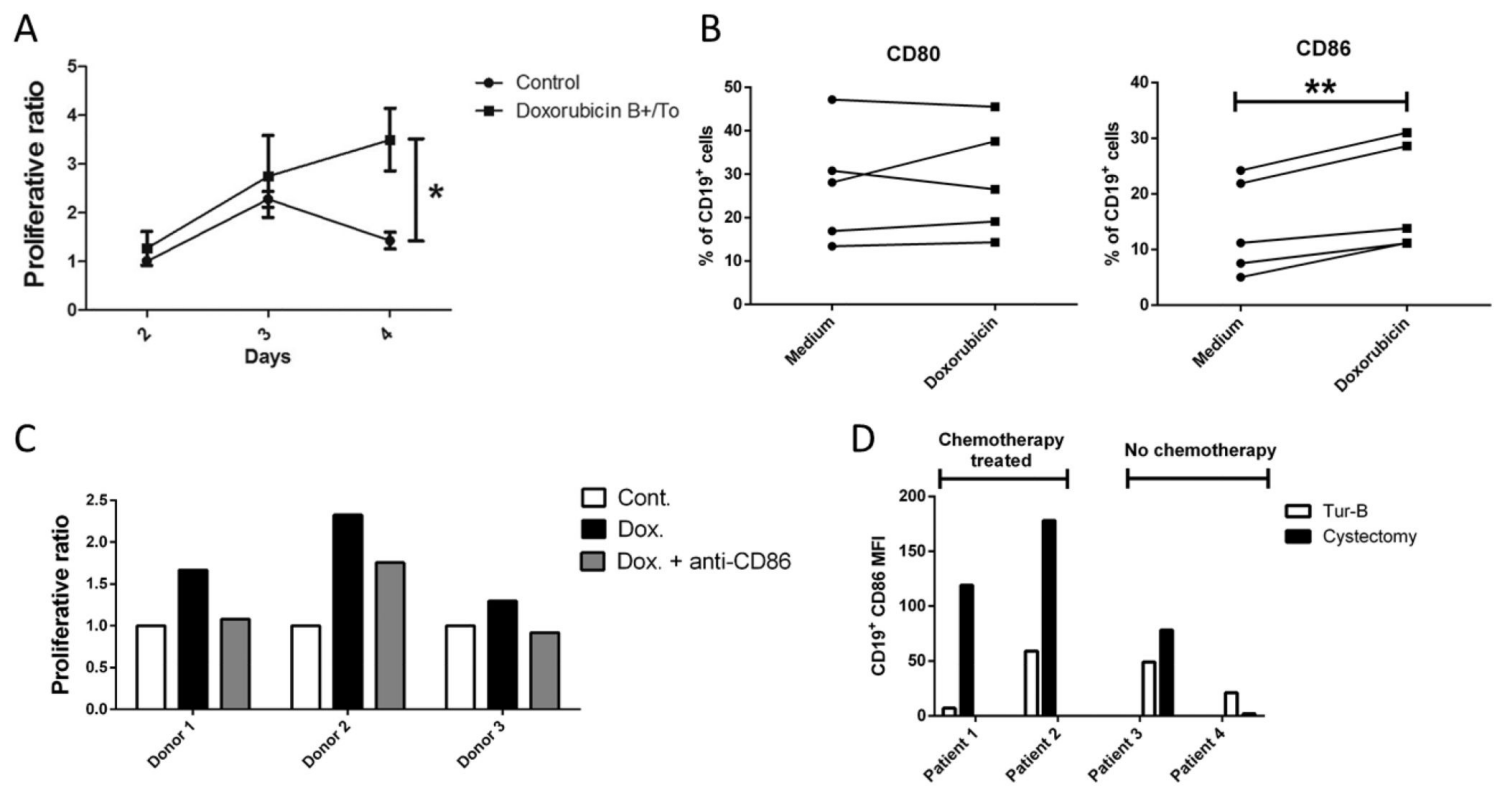
Doxorubicin treatment upregulates CD86 expression on B cells and increases the proliferation of CD4^+^ T cells. A) Human CD4^+^ T cells and CD19^+^ B cells from healthy donors were co-cultured and an increased in CD4^+^ T cell proliferation was observed with doxorubicin treatment. B) Human CD19^+^ lymphocytes were isolated from PBMCs and treated with doxorubicin, which increased CD86 expression but did not alter CD80 expression. C) Doxorubicin-treated B cells incubated with CD86-blocking antibody (gray) showed a decrease capacity to activate co-cultured CD4^+^ T-cells. D) PBMCs from two muscle-invasive urinary cancer patients who received chemotherapy (Patient 1 and Patient 2) and two patients who did not receive neoadjuvant chemotherapy (Patient 3 and Patient 4) were compared. CD86 expression on CD19^+^ cells was correlated to the level of PBMCs.^[138]^ Copyright 2008, Elsevier.

B cells are increasingly considered as a crucial component in antitumor immunity, because of antigen presentation and cancer antibody production after differentiation into plasma cells.^[130,131]^ Tumor-infiltrating B cells (TIL-B) have been found to be important for cancer immunotherapy, ^[132–134]^ contributing to the formation of CD4^+^ memory T cells and promoting the survival and proliferation of CD8^+^ T cells. Evidence for the positive impact of chemotherapeutic agents on antitumor immune response also arises from activating B cells.^[135]^ Erik et al. found that administration of cyclophosphamide not only increased the percentage of CD3^+^ and CD4^+^/CD8^+^ T cells but also improved the percentage of B cells in the spleen and tumor.^[136]^ Additionally, several experiments indicated that incubation of B cells with low levels of doxorubicin led to increased proliferative B cell response, which was attributed to the upregulation of the co-stimulatory molecule CD86.^[137]^ Winqvist et al. investigated the influence of cisplatin, doxorubicin and irinotecan on the antigen-presenting ability of B cells.^[138]^ Pre-treatment of B cells with irinotecan and cisplatin showed moderate increase in CD4^+^ lymphoblast formation when co-cultured with untreated CD4^+^ T cells. Interestingly, when doxorubicin treated B cells were used as antigen-presenting cells, there was a significant increase in lymphoblast formation (Figure 7A). The expression of HLA-DR (MHC-II cell surface receptor) on CD19^+^ B cells remained unchanged when cultured with doxorubicin. However, doxorubicin treatment led to an increased expression of CD86, while CD80 expression remained unaltered (Figure 7B). These data suggest that doxorubicin-enhanced antigen presentation by B cells was achieved by amplifying the CD86 co-stimulatory signal. This hypothesis was confirmed by CD86 blocking which reduced blast transformation of CD4^+^ T cells (Figure 7C). In patients treated with chemotherapy, PBMCs were significantly increased when CD86 expression on CD19^+^ B cells was upregulated (Figure 7D). These findings suggest that chemotherapy may have positive immunomodulatory effects in anti-tumor immune response through B cell activation.

## 4 Conclusion

Numerous preclinical and clinical studies have demonstrated that, although high-dose chemotherapy generally results in immunosuppression, chemotherapeutic drugs can also directly stimulate immunity, especially when administered at noncytotoxic doses. Hence, understanding the direct impact of chemotherapeutics on immune cells is crucial for optimizing cancer treatment strategies and improving patient outcomes. One of the key effects of chemotherapeutic agents is the promotion of immune cell maturation. Such direct immunostimulation is different from the so-called immunogenic cell death which indirectly stimulates immunity by danger-associated molecular patterns released by apoptotic cancer cells. For example, several drugs can induce the maturation of DCs; these drugs include clinically well-used taxanes. This enables the effective presentation of cancer cell antigens to T cells, leading to a robust and specific antitumor immune response. Chemotherapeutic drugs also can directly contribute to lymphocyte activation. The immunostimulation role of chemotherapeutics opens new avenues for combination therapies that aim to synergize the cytotoxic effects of chemotherapy with its immunostimulatory potential, ultimately leading to more effective cancer treatment strategies. Further research efforts are warranted to unravel the underlying mechanisms governing these effects and to develop innovative approaches that enhance the anti-tumor response while minimizing immunosuppression. One promising approach lies in the formulation of chemotherapeutic drugs in nanomedicines to enable targeted delivery and controlled release, as well as decreased side effects and immunosuppression. By combining chemotherapy and immunotherapy, with a focus on harnessing chemotherapy-induced direct immune cell activation, we envision improved therapeutic outcomes and enhanced quality of life for cancer patients.
